# Spectrally resolved X-ray scattering

**DOI:** 10.1039/d6fd00068a

**Published:** 2026-05-26

**Authors:** David J. Romano, Benjamin H. Savitzky, Andres Moreno Carrascosa, Alexander Narkiewicz-Jodko, Joseph D. Geiser, Patrick Yuheng Wang, Lisa Huang, Stuart W. Crane, Xinxin Cheng, Mengning Liang, Sandra Mous, Michael P. Minitti, Mats Simmermacher, Adam Kirrander, Peter M. Weber

**Affiliations:** a Department of Chemistry, Brown University Providence RI 02912 USA peter_weber@brown.edu; b Department of Chemistry, University of Oxford Oxford OX1 3QZ UK adam.kirrander@chem.ox.ac.uk; c Linac Coherent Light Source, SLAC National Accelerator Laboratory Menlo Park CA 94025 USA

## Abstract

The Bethe surface represents the X-ray scattering cross section as a function of momentum transfer and energy loss, mapping the electronic spectrum of the target *via* the inelastic transition matrix elements. By effectively merging spectroscopy and scattering, a time-resolved measurement of the molecular Bethe surface during a chemical reaction could reveal details of the reaction path through the manifolds of electronic states and conical intersections. However, conventional imaging detectors used for X-ray scattering lack energy resolution. We demonstrate here how the Bethe surface can be measured with the help of a spectral filter in the form of a thin zinc metal foil inserted between the target and the detector. Harnessing the inherent stochasticity of X-ray pulses generated by self-amplified spontaneous emission at X-ray free-electron lasers (XFELs), we demonstrate how the double-differential X-ray scattering cross section can be reconstructed using a novel ghost-imaging algorithm. Corrections for fluorescence from the metal filter need to be applied. The method requires excellent signal levels that can be obtained for molecules with large scattering cross sections, but advances in XFEL technology promise opportunities for a wide range of applications.

## Abstract 2

Ghost-imaging with stochastic XFEL pulses and a zinc spectral filter enables reconstruction of the Bethe surface of gas-phase SF_6_, bridging scattering and spectroscopy on a single imaging detector.

## Graphical abstract

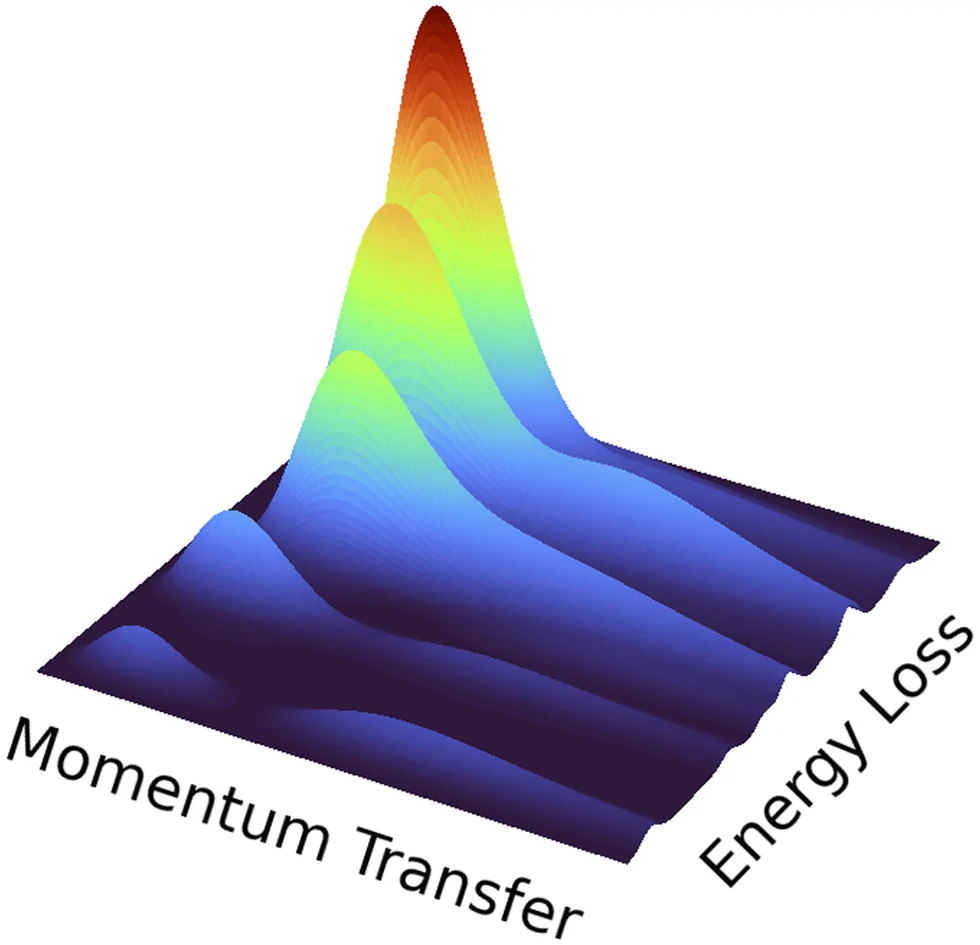

## 1 Introduction

The study of gas-phase molecular structure and dynamics has been profoundly advanced by the emergence of X-ray free-electron lasers (XFELs).^1–3^ These novel instruments now enable time-resolved gas-phase experiments, achieving excellent spatial and temporal resolution with high signal-to-noise ratios. X-ray scattering experiments have observed molecular evolution on ultrashort time scales upon optical excitation, revealing subtle electronic and nuclear effects.^4–6^ Recent developments also include the measurement of intramolecular charge transfer, the observation of nuclear dynamics, and determination of the orientation of transition dipole moment.^7–13^ Conventional imaging detectors lack energy resolution and conflate elastic and inelastic scattering. This detector-level energy integration obscures subtle effects of electronic structure on the scattering signal, effectively hiding energy-resolved observables. We will present in this paper an approach for retrieving such information using existing energy-integrating detectors. Non-resonant X-ray scattering can be expressed in terms of a double-differential X-ray scattering cross section (DDSCS). The DDSCS depends on the solid angle *Ω* and the energy loss Δ*E* = *E*_i_ − *E*_s_, obtained as the energy difference between the initial and the scattered photon. Within the Waller–Hartree approximation, the DDSCS is expressed as^14–16^



1

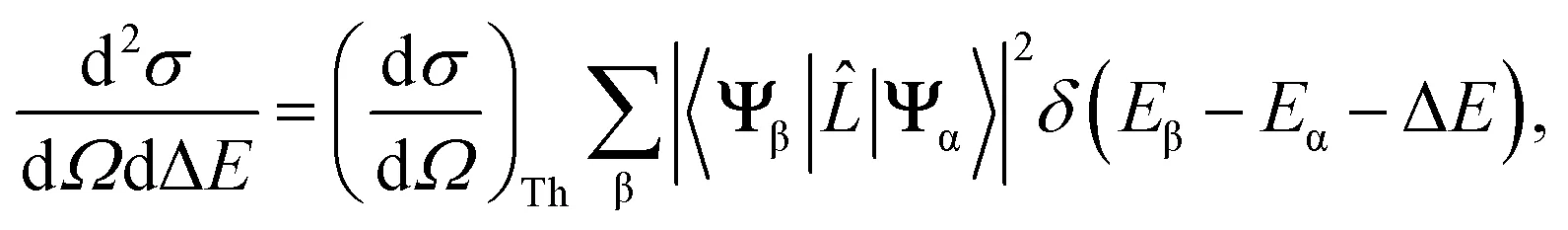

with (d*σ*/d*Ω*)_Th_ the Thomson scattering cross section of a free electron,



2

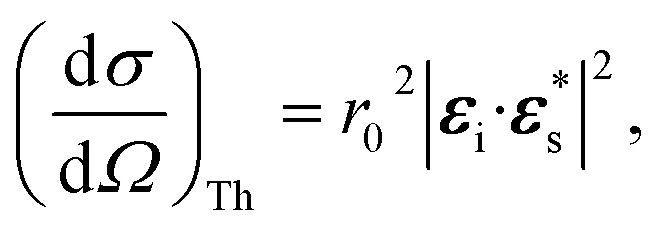

where *r*_0_ is the classical electron radius and 
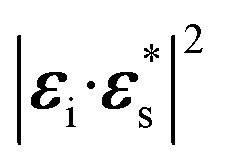
 is the polarization factor for the incident and scattered radiation. Returning to eqn (1), **Ψ**_α_ and **Ψ**_β_ are the initial and final states with their respective energies *E*_α_ and *E*_β_. *L̂* is the scattering operator,3
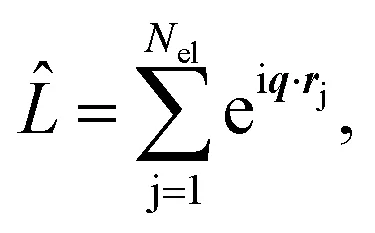
with the sum running over all *N*_el_ electrons j, and the momentum transfer vector ***q*** = ***k***_i_ − ***k***_s_ is the difference between the incident and scattered wave vectors.

Gas-phase X-ray scattering experiments at XFELs are performed using energy-integrating area detectors that record the total scattering d*σ*/d*Ω* only.^17^ This total X-ray scattering involves a sum over all possible transitions between the initial and final electronic states. Based on the completeness of the sum over states, this then reduces to a measurement of the initial state only.^14^ This is not necessarily bad, and as discussed above, a wide range of important and highly relevant information can be retrieved from such experiments.^18–20^ At the same time, it is clear that highly valuable information about excited states is lost in the process.

This information would be available from spectrally resolved X-ray scattering measurements, where the individual inelastic components that contribute to the total X-ray scattering signal are isolated and characterized as a function of the momentum transfer *q* and the energy loss Δ*E*. This spectrally resolved scattering signal would provide deeper insight into the molecular electronic landscape and bridge the gap between scattering and spectroscopy. Experimental implementations that record X-ray scattering patterns with spectral resolution would therefore mark a major advance in scattering methodology.

Fig. 1 illustrates this theoretically for the water molecule by graphing the scattering signal as a function of energy loss and the absolute value of the momentum transfer vector in what is called a Bethe surface.^21^ With an assumed energy resolution of 0.56 eV, it includes the inelastic X-ray scattering signals of the first 20 singlet excited states of H_2_O, calculated at the second-order algebraic diagrammatic construction level of theory^22^ with the augmented correlation-consistent polarized valence double-zeta basis set^23,24^ supplemented with Rydberg functions from Kaufmann *et al.*,^25^ hereafter denoted ADC(2)/aug-cc-pVDZ+Rydberg. The distinct features of each transition are immediately apparent. The magnitude of these inelastic X-ray signals is proportional to the generalized oscillator strength of the excitation,^26^ while the positions of the maxima are closely related to the orbital overlap dominating the transition.

**Fig. 1:**
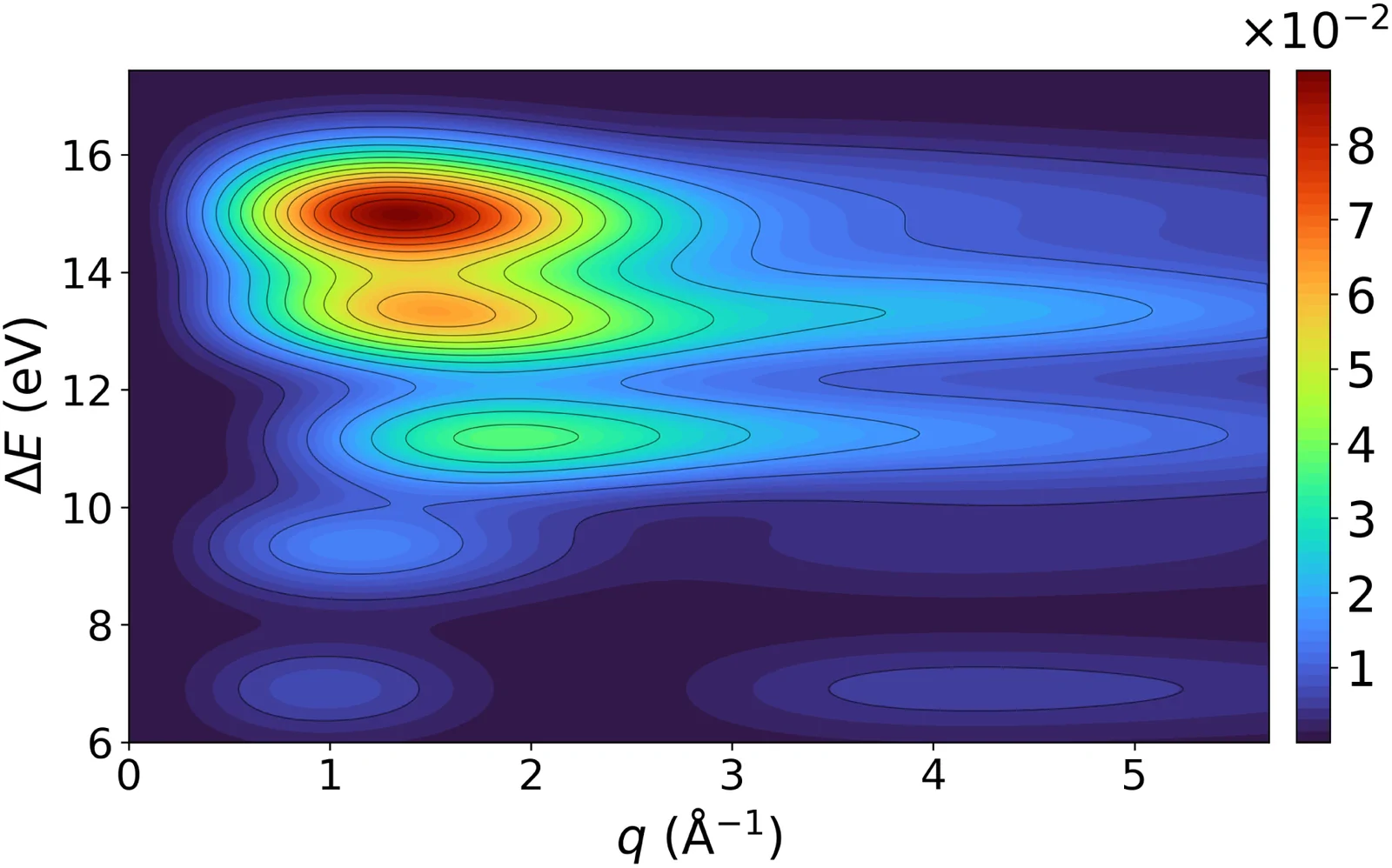
Theoretical Bethe surface of the water molecule. Calculated Bethe surface for the water molecule H_2_O depicting the first 20 inelastic scattering transitions calculated with ADC(2)/aug-cc-pVDZ+Rydberg.

Full characterization of the Bethe surface requires spectrally separating these components and has been historically achieved with energy-resolving detectors with limited solid-angle coverage^27^ or indirect estimation using independent-atom model (IAM) calculations to approximate and subtract the inelastic background.^28^ Neither approach is fully satisfactory: the former sacrifices counting statistics and abandons *q* resolution, while the latter presupposes the very electronic structure one wishes to measure. Most early investigations of the Bethe surface have therefore employed electron scattering,^29^ even though it remains difficult to spectrally analyze the electron energy spectrum with modern imaging detectors.

In this work, we present a new method for the direct, model-independent, experimental spectral separation of total X-ray scattering signals, which we implemented at the Linac Coherent Light Source (LCLS). By developing a spectral ghost-imaging technique, we exploit the intrinsic shot-to-shot spectral stochasticity of self-amplified spontaneous emission (SASE) pulses in conjunction with a thin zinc foil acting as a spectral filter. Correlation of the measured scattering intensities with independently recorded pulse spectra allows reconstruction of the energy-resolved scattering profile from measurements made with a conventional energy-integrating detector. Here we describe the experimental and analytical methodology, including preprocessing, correction for fluorescence from the zinc foil, and the ghost-imaging spectral signal inversion, and demonstrate successful application of the technique on gas-phase SF_6_.

## 2 Methods

### 2.1 Experimental details

The linearly polarized 120 Hz, ∼25 fs (FWHM), ∼1.5 mJ primary beam of the LCLS with a nominal photon energy of 9.666 keV was focused using Kirkpatrick–Baez mirrors into a windowless gas flow cell with a beryllium out-coupling window that minimized absorption and re-scattering. The gas-phase sample of sulfur hexafluoride was held at ∼20 Torr. A 15 µm thick sheet of zinc foil was placed ∼3.8 mm after the beryllium window normal to the primary beam, occluding about half of the detector. The scattered X-rays were detected with a Jungfrau-4M hard X-ray detector with a pixel size of 75 µm × 75 µm situated ∼9.5 cm behind the scattering cell. In addition, before entering the scattering cell, a small fraction of the primary beam was directed to a bent-crystal hard X-ray single-shot spectrometer (HXSSS) using the Si(110) crystal for a (440) reflection.^30,31^ Shortly upstream of the scattering cell, the primary beam was directed through a thin SiN foil. The intensity of the Compton back-scattering from this foil was measured *via* 8 diodes. This setup, known as the intensity position monitor (IPM), was used as an in-line nondestructive measurement of the number of photons in each pulse.^32^ The experimental setup is depicted in Fig. 2.

**Fig. 2:**
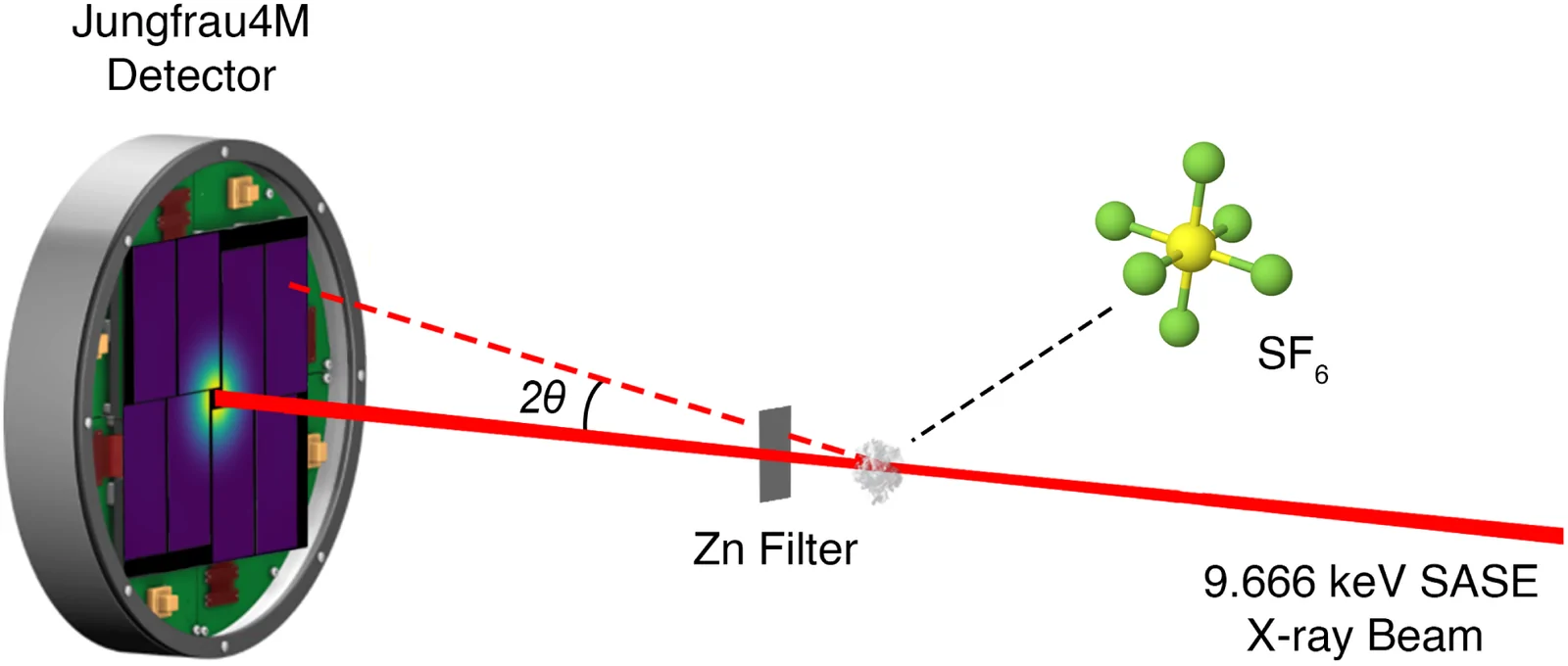
Experimental setup. SASE X-ray pulses from the LCLS are focused onto a gas-phase sample of sulfur hexafluoride, SF_6_. A zinc foil is placed in front of one side of the scattering cell, filtering half of the post-specimen scattered photons before they hit the detector. The signals on both the filtered and unfiltered sides are recorded with a Jungfrau-4M detector.

### 2.2 Preprocessing

Two-dimensional scattering patterns from the Jungfrau-4M detector were recorded on a shot-by-shot basis. Each image was gain-corrected to a readout in analog-to-digital units (ADU), closely related to the X-ray photon energy. For each image, the ADU readings were thresholded to remove dark counts, sample fluorescence, and other spurious signals while preserving the integrity of the scattering data. A mask was applied to each image to remove overactive or bad pixels from the analysis. For this experiment, the mask removed ∼6% of all pixels.

The high-intensity X-ray pulses were excluded from the calibration steps to remove absorption-scattering events by setting the threshold based on deviation from kinematical theory. The unfiltered portion of the two-dimensional scattering pattern was used to determine the detector position by fitting a theoretical total scattering pattern of SF_6_, calculated with in-house scattering code^18,19,33^ at the coupled-cluster singles and doubles level of theory^34^ with the augmented correlation-consistent polarized valence double-zeta basis set, CCSD/aug-cc-pVDZ. The fitting process iteratively accounts for the correction factors addressed in ref. 17, the variation of the Thomson scattering cross section with respect to the X-ray polarization, and physical geometry of the Jungfrau-4M detector. Once the optimal detector center and distance were found, each image was divided into the unfiltered and filtered portions, azimuthally averaged, and transformed into momentum space to produce two one-dimensional scattering profiles for each shot.

Using the data from the IPM, HXSSS, and the filtered and unfiltered azimuthally averaged scattering signals, specific shots were removed to eliminate incomplete or damaged data.

### 2.3 Accounting for zinc fluorescence and shot-by-shot variations

The experimental setup for spectral ghost-imaging requires that a metal foil be placed in front of the main scattering cell with the specific purpose of blocking a fraction of the scattering as a function of photon energy. However, some of the absorbed photons may be re-emitted as fluorescence, which may impinge on the detector. In our case, the zinc *K*_α_ and *K*_β_ fluorescence emission lines are not removed by the ADU threshold and add spurious signal. Therefore, it is necessary to determine and correct for the impact of the fluorescence signal on the final result. To do so, we first calculate the intensity of the scattered radiation on the zinc foil and then evaluate the amount of fluorescence from each pixel element of the foil. The fluorescence intensity measured by the detector is then obtained by summing the emission contributions from all pixel elements of the metal foil.

We start with an *ab initio* scattering pattern calculated *via* our in-house code.^18^ This software produces a one-dimensional differential scattering pattern d*σ*/d*Ω*. In this form, the total differential X-ray scattering cross section has been normalized by the Thomson scattering cross section for an electron *σ*_Th_. Therefore, the units of d*σ*/d*Ω* are inverse steradians, sr^−1^.

To determine the scattering occurring in a given pixel element on a surface normal to the optical axis, the *ab initio* differential cross section must be multiplied by a prefactor d*Ω* that converts the cross section per unit solid angle into the cross section per pixel element while accounting for off-axis pixels' reduced effective cross sections and for the inverse-square law intensity dependence. This prefactor is given by4
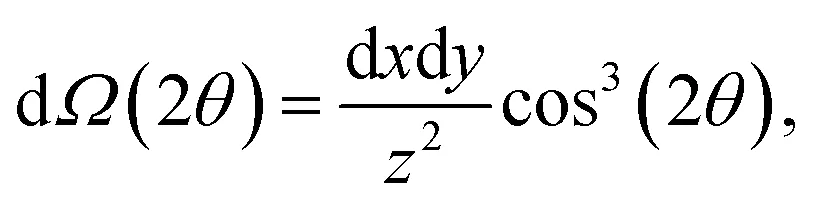
where d*x* and d*y* are the pixel's *x* and *y* extents, *z* is the normal distance from the source to the surface, and 2*θ* is the polar angle of scattering relative to the incident beam.

Further, the incident beam is linearly polarized, requiring an additional *ϕ*-dependent polarization factor,

5 *C*_Th_(2*θ*,*ϕ*) = sin^2^(*ϕ*) + cos^2^(2*θ*)cos^2^(*ϕ*),

where *ϕ* is the azimuthal angle relative to the photon polarization. Combining these together, the exposure of a zinc pixel with coordinate (2*θ*, *ϕ*), area d*x*d*y*, normal distance *z*, and incident photon energy *E*_i_ is given by the effective cross section6
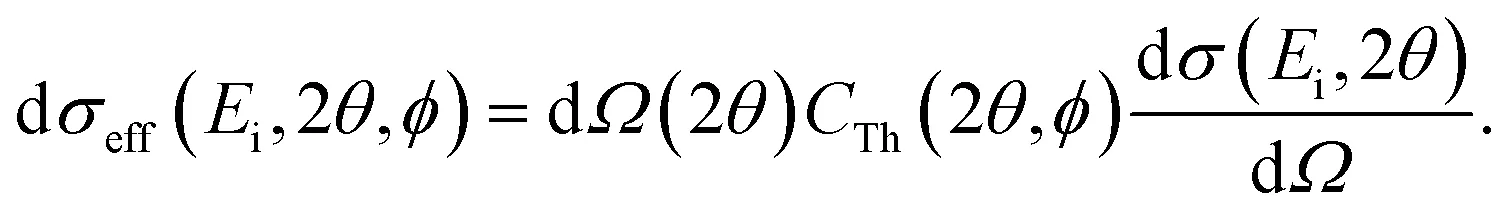


Fluorescence by itself is a completely isotropic emission process. However, one can imagine a situation where the absorption and fluorescence happen at the very beginning of the zinc foil; then, the fluoresced light must travel through the rest of the foil to reach the detector. As the path length through the foil is different for every fluorescence polar angle *θ*′, differing fractions of fluoresced light will be reabsorbed as a function of that angle, therefore the resultant “fluorescence pattern” becomes anisotropic in *θ*′. To determine the fluorescence pattern emitted by each normal pixel element on the zinc foil, we start by calculating the amount of light incident on a length element d*L* at depth *L* through the zinc, where both d*L* and *L* are normal to the surface of the zinc foil. This process follows the Lambert–Beer law:7
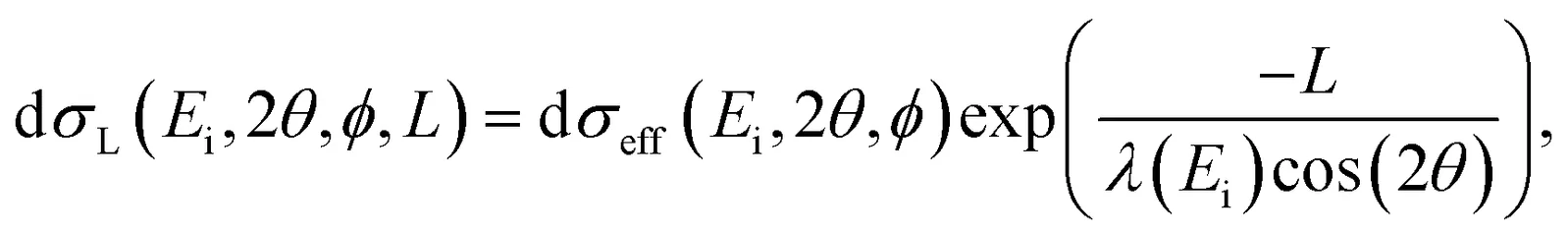
where *λ*(*E*) is the attenuation length of the zinc at energy *E*. The amount of light absorbed through the length element d*L* is the negative rate of change of d*σ*_L_(*E*_i_, 2*θ*, *ϕ*, *L*) with respect to *L*. A fraction of this absorbed light, *η*, will be fluoresced. This fraction is found using the following formula,

8 *η* = *ω*_K_*J*_K_ ≈ 0.422,

where *ω*_K_ is the K-shell fluorescence yield and *J*_K_ is the K-shell jump factor for zinc. Only the K-shell is considered here as less energetic fluorescence emissions are filtered by the ADU threshold of the Jungfrau-4M detector. The fluoresced light is spread over 4π steradians, and attenuated by the length of zinc it passes before traveling to the detector. This remaining distance depends on the fluorescence polar angle *θ*′, but is isotropic in the fluorescence azimuthal angle *ϕ*′. Therefore, the differential fluorescence pattern for a given length element d*L* inside a pixel (2*θ*, *ϕ*) is9
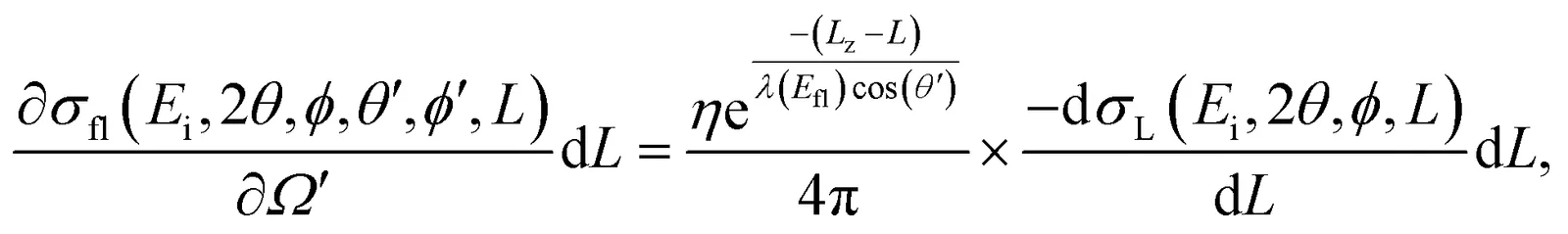
where *L*_z_ is the thickness of the zinc foil, and *E*_fl_ is the fluorescence photon energy. Finally this term is then integrated over all possible fluorescence depths within the zinc foil to give an expression for the total differential fluorescence pattern emanating from a given (*x*, *y*) position on the zinc foil,10
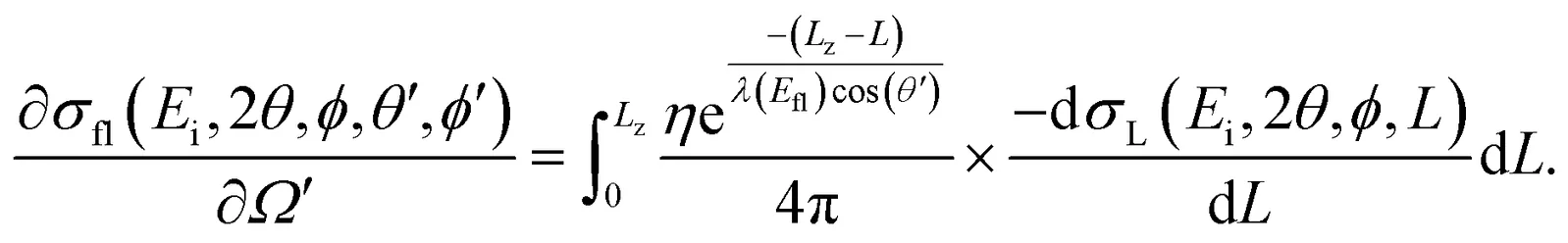
This integral can be solved analytically, giving the final expression,11
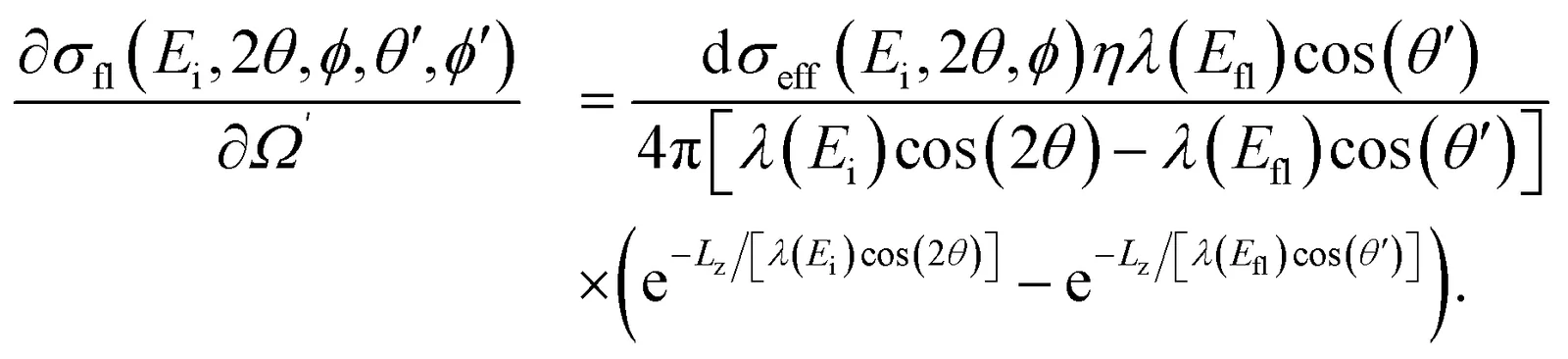


To find the total accumulation of fluorescence on the detector, the zinc foil is discretized into pixels in the normal direction, eqn (11) is calculated for each of these zinc pixels, and the output fluorescence is propagated from each zinc pixel to the detector. There is no polarization factor in this case as the fluoresced light is unpolarized. The total fluorescence on a given detector pixel is then12
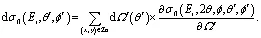


Note that the prefactor d*Ω*′ here differs from the prefactor d*Ω* in eqn (6). In eqn (6), d*Ω* is evaluated with eqn (4) using d*x*d*y* as the pixel size chosen to discretize the zinc foil, and *z* as the normal distance from the scattering cell to the zinc foil. Here, when eqn (4) is used to determine d*Ω*′, d*x*d*y* is the pixel size on the detector, and *z* is the normal distance from the zinc foil to the detector.

This entire process thus far assumes that the scattering signal originates from a single point. However, there is a roughly 2 mm long scattering volume, and many of the geometric parameters change when the scattering source location is changed. Therefore, the signal must be integrated across the entire scattering length. The final amount of incident fluorescence intensity with respect to the simulated scattering intensities is shown in Fig. 3.

**Fig. 3:**
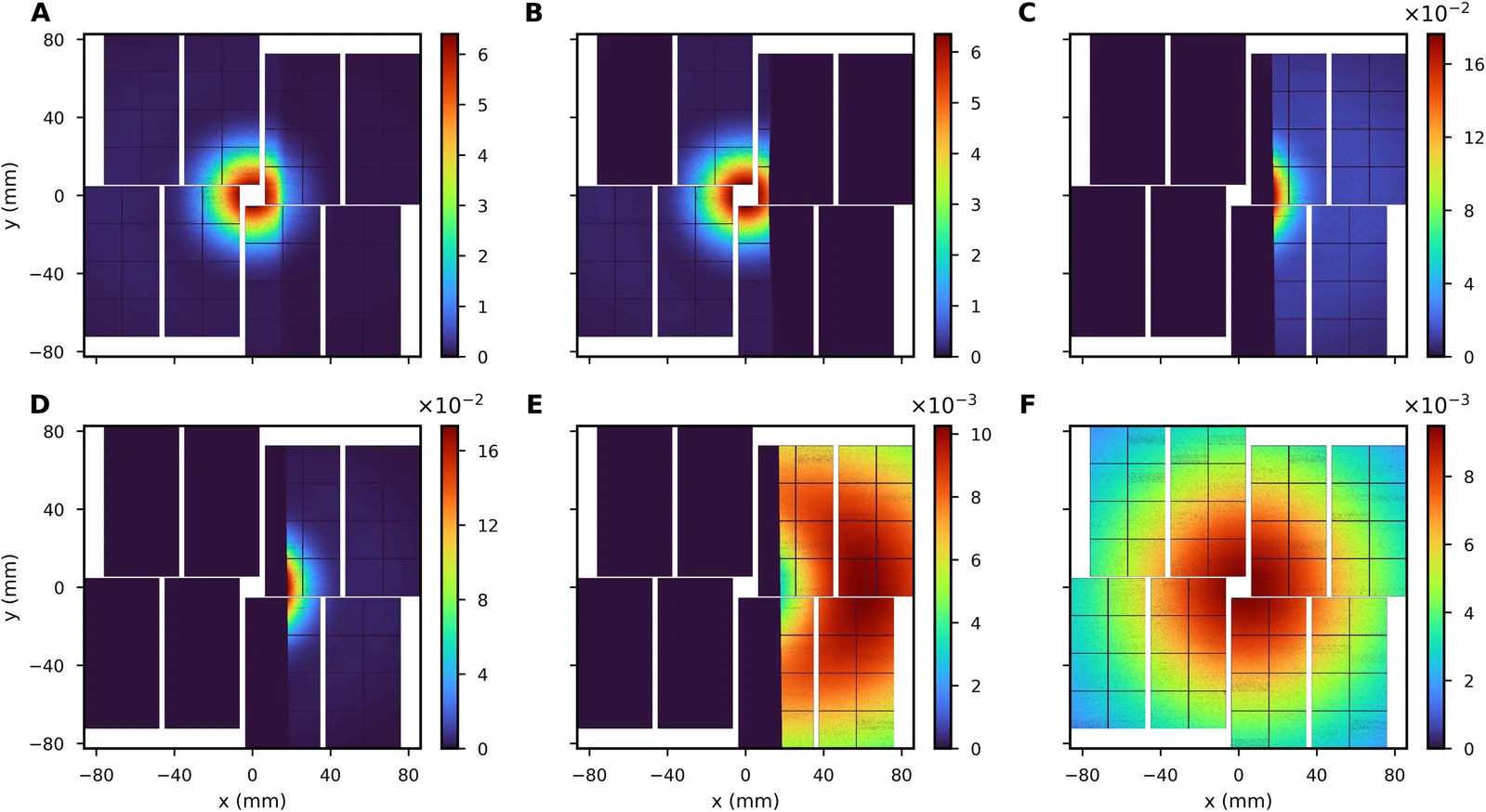
Scattering of SF_6_ and Zn fluorescence. (A) Experimental scattering. Filtered region of the detector is seen on the right-hand side. (B) Simulated unfiltered total scattering. (C) Simulated filtered total scattering. (D) Simulated filtered elastic scattering. (E) Simulated filtered inelastic scattering. (F) Simulated zinc K-shell fluorescence. The color scale is in arbitrary intensity units and masked pixels are represented as 0.

To determine the effect of this fluorescence for the unfiltered and filtered azimuthal averages on any given shot with spectrum *m*(*E*_i_), ratios were taken from these simulation results of the azimuthal average of the filtered scattering signal to the azimuthal average of the filtered scattering with fluorescence, and likewise for the unfiltered signal. These two curves are shown in Fig. 4. Fluorescence from the K-shell can occur only if the incident photon energy is above the zinc K-edge. The parts of the SASE pulse with energies below the K-edge will not produce fluorescence. Therefore, correction matrices were created containing the fluorescence ratio curves above the K-edge, and unity everywhere else. Dot products of the correction matrices were taken with the incident spectra to determine a shot-to-shot correction factor for the filtered and unfiltered azimuthally averaged signals. These correction factors were then multiplied element-wise to the shot-to-shot azimuthally averaged signals before proceeding with the ghost-imaging step of the analysis.

**Fig. 4:**
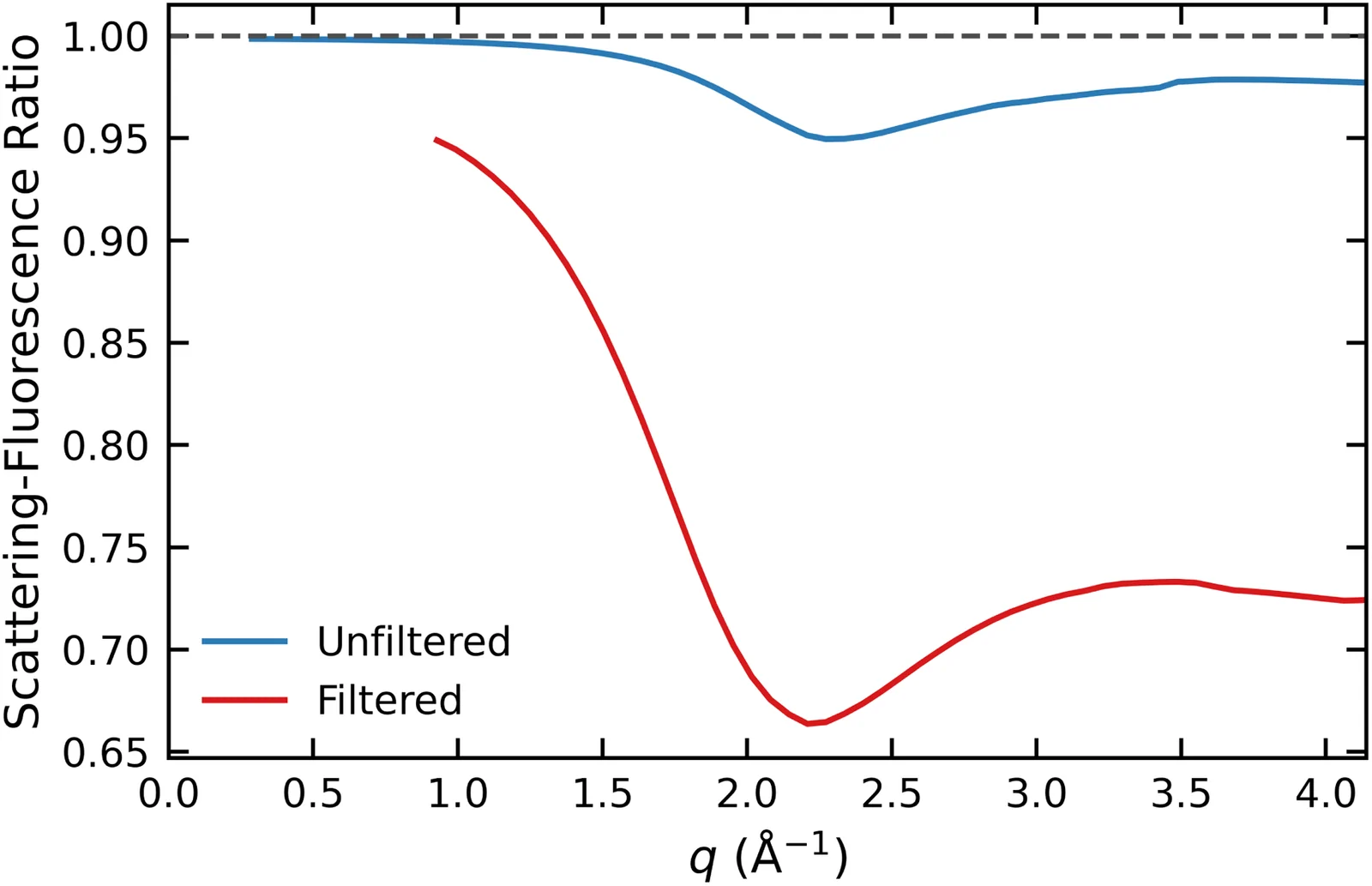
Zinc fluorescence ratios. For the unfiltered and filtered sides of the detector, the scattered light is expressed as a fraction of the total signal (scattering and fluorescence from the zinc foil) incident on the detector. The filtered portion does not extend as low in *q* as the unfiltered curve due to the geometry of the Zn filter.

## 3 Results and discussion

### 3.1 Spectral ghost-imaging

Ghost-imaging, which was originally developed for quantum systems in the spatial domain,^35–45^ correlates a beam passing through an object of study and measured on a “bucket” (*i.e*. integrating) detector that has no spatial resolution with a reference beam that is measured with spatial resolution but never encounters the object. The general ghost-imaging concept has been mapped onto other domains, for example the time domain to resolve ultrafast time-dependent processes.^46^ Here, we adapt the scheme to the energy domain in order to spectrally resolve the total scattering signal by taking a pre-sample shot-by-shot measurement of the X-ray spectrum as a reference signal, while using each pixel of the imaging detector as a bucket detector with no resolution in energy.

The spectral decomposition of the scattering signal exploits stochastic fluctuations of the SASE pulses from the LCLS XFEL. On a shot-by-shot basis, the spectra of the X-ray pulses fluctuate over a ∼25 eV (FWHM) range as illustrated in Fig. 5. For each shot j, the measured scattering intensity *d*_j_(*q*) is given by13
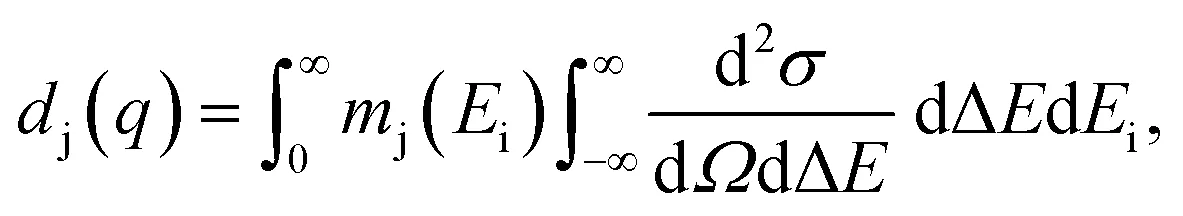
where *m*_j_(*E*_i_) is the energy spectrum of incident pulse j. The integral over Δ*E* reflects that the detector lacks energy resolution. Note that the DDSCS and ghost-imaging framework are formulated in terms of the momentum transfer *q*, as this dictates the intrinsic scattering physics of the sample and is the natural description in the molecular frame. However, the experimental data is measured in the laboratory frame at fixed polar, 2*θ,* and azimuthal, *ϕ*, scattering angles. As discussed in Section 2.3, the spurious fluorescence signals are also highly dependent on the experimental geometry. Therefore, the fluorescence corrections and pre processing are carried out in the laboratory frame. Once these artifacts are corrected, the signals are cast onto a common *q*-grid *via* the relation *q* = (4π/*λ*) sin  *θ* before proceeding with the ghost-imaging analysis.

**Fig. 5:**
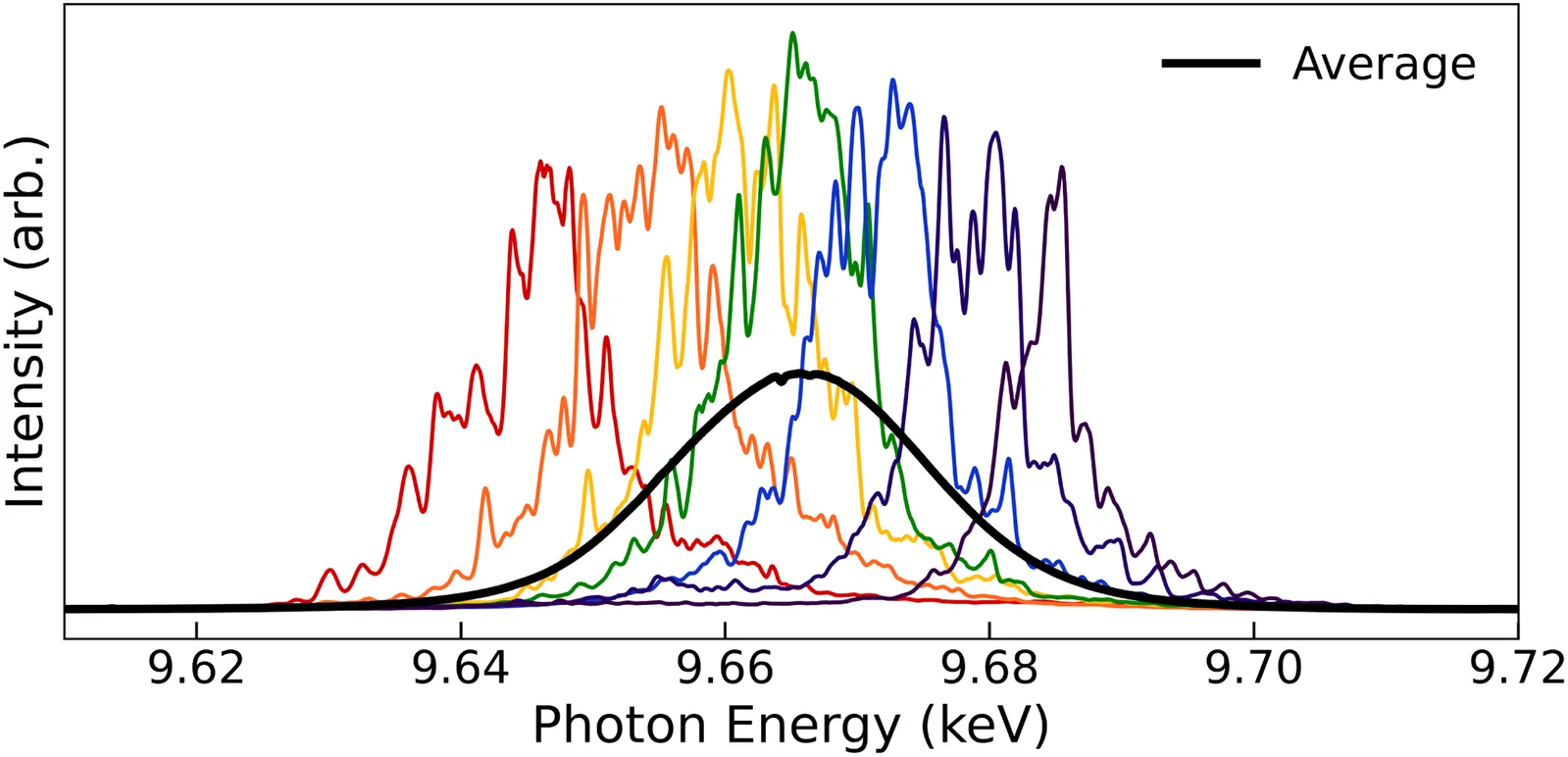
LCLS X-ray spectra. Seven representative spectra of individual SASE pulses. The average spectrum of all pulses in the dataset is shown in black. The spectral incoherency and stochastic fluctuations enable the reconstruction of the inelastic and elastic X-ray scattering by ghost-imaging.

Importantly, the integral over Δ*E* in eqn (13) destroys the energy-dependent information, such that spectral separation of the total scattering is no longer possible in a traditional diffraction experiment. To apply our ghost-imaging method for recovering this information, the zinc foil that covers approximately half of the scattering detector serves as a spectral filter. The center of the incident photon energy is tuned just above the Zn K-edge, such that the filter transmission *f*(*E*_s_) is large for photons with Δ*E* > 0 and small otherwise. Incident X-ray pulse spectra *m*_j_(*E*_i_) are measured with the pre-scattering cell HXSSS alongside the scattering signal on the unfiltered and Zn-filtered halves of the detector, yielding a pulse energy spectrum and two complementary scattering (half) images for each shot.

In the presence of the Zn filter, the scattering intensity in eqn (13) becomes14
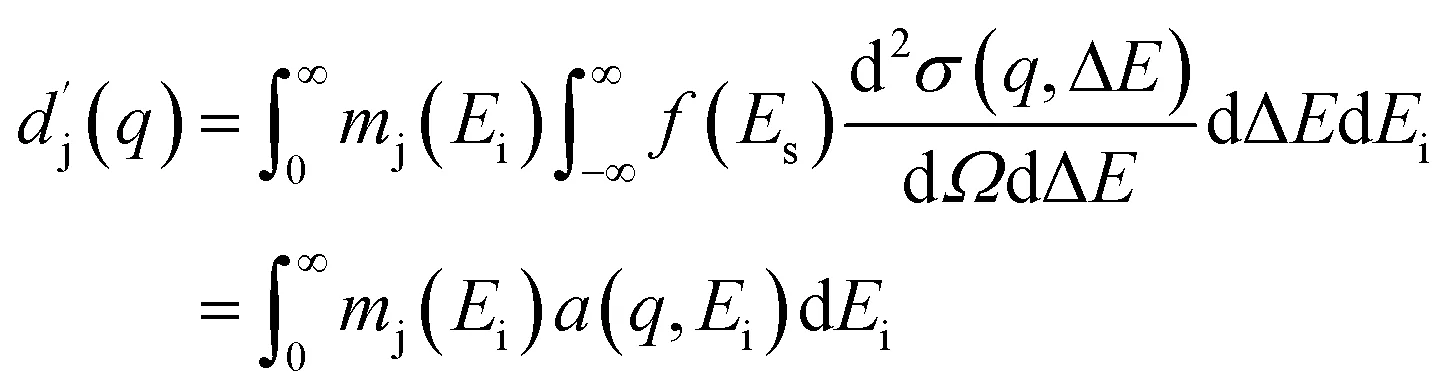
where 
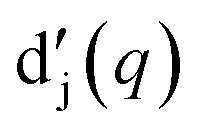
 is the filtered scattering intensity and 15
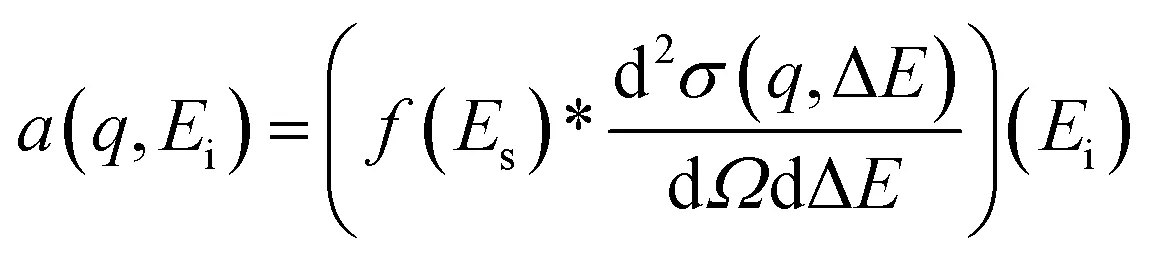
is the dynamic structure factor convolved in energy with the filter function. In the absence of a filter, the filter function reduces to *f*(*E*_s_) = 1 and we revert to eqn (13).

The ghost-imaged object is the quantity *a*(*q*, *E*_i_). To recover *a*(*q*, *E*_i_), we divide the momentum-transfer range into small *q*-bins and treat each bin as a bucket, then extract *a*(*q*_0_, *E*_i_) for every discrete value of *q*_0_. With a large number of measurements *N*_j_ and with *N*_*ε*_ energy bins, we define 
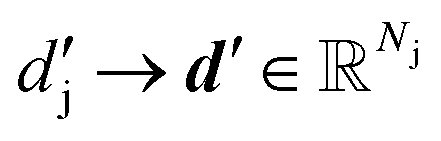
, 
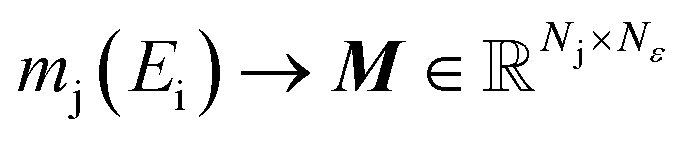
, and 
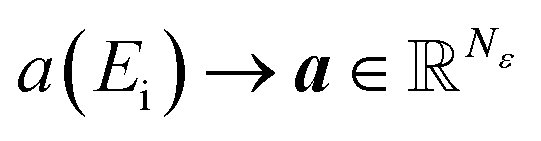
, and form the equation

16 ***d*′** = ***M·a***,

which is the discretized form of eqn (14) taken over all shots *j* = 1, …, *N*_*j*_. Provided sufficient signal levels, eqn (16) can be inverted to retrieve ***a*** over the energy range sampled by the stochastic SASE pulses.

In practice, shot-to-shot intensity variations as well as slower variations in the gas flow (and thus sample density) will impart artifacts in the spectral correlation when determining ***a***. These variations must therefore be removed before proceeding with the ghost-imaging analysis. To do so, the ratio of the filtered to unfiltered azimuthally averaged scattering was used in place of ***d***′ in the ghost-imaging analysis. Simply put, the final quantities ***a*** and by extension the DDSCS are expressed as a ratio to the energy-integrated total scattering, d*σ*/d*Ω*.

### 3.2 Recovering the double-differential X-ray scattering cross section

The recorded spectra were down-sampled to an energy pixel resolution of 0.56 eV and area-normalized to unity to preserve the correlation to the scattering ratio. Using the scattering ratio of filtered to unfiltered azimuthal averages and the area-normalized spectra discussed previously, a ridge regression was used to solve for ***a*** with *λ* = 1.5 to mitigate nonphysical regression artifacts. Fig. 6 shows the result of the spectral ghost-imaging process for the range of scattering angles. Since the dominant portion of the scattering signal stems from the elastic scattering, Δ*E* = 0, the primary feature in Fig. 6 is a signature of the zinc K-edge itself. *q*-dependent features are evident above the convolved edge, consistent with inelastic scattering contributions to the signal.

**Fig. 6:**
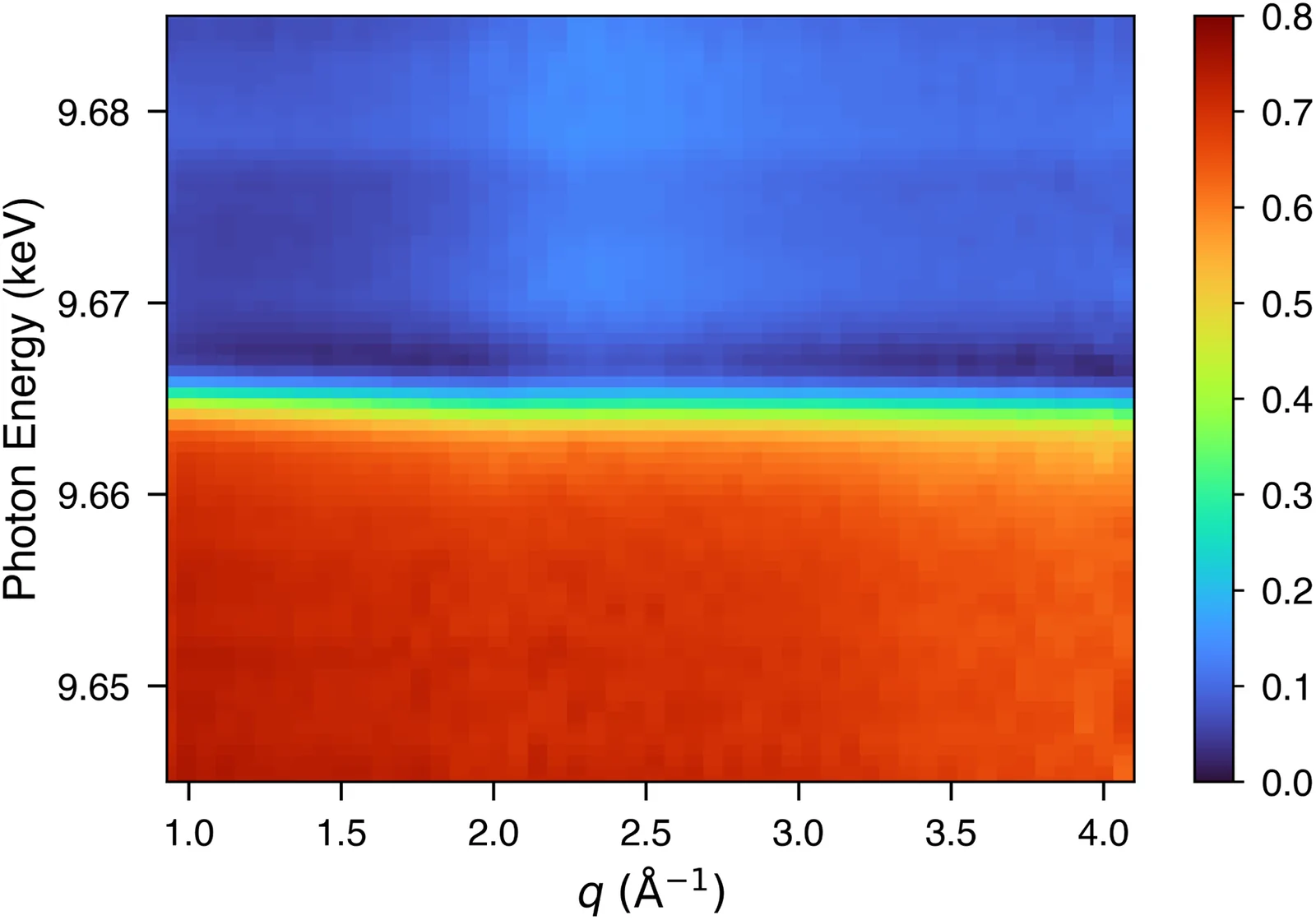
Ghost-imaged ***a***-vectors. The edge-shaped feature at 9.665 keV is the impression of the zinc K-edge and the elastic scattering. *q*-dependent features exist above the edge, indicative of inelastic scattering. Due to the ratio of scattering used in the ghost-imaging analysis, the values of ***a*** are unitless.

The ghost-imaged quantity, ***a***, represents the dynamic structure factor convolved with the zinc absorption spectrum. Therefore, if the filter function *f*(*E*_s_) is known, ***a*** can be deconvolved to determine the DDSCS. As the inelastic X-ray scattering signal tends to zero at low momentum transfer, we can recover *f*(*E*_s_) from *a*(*q*, *E*_i_) by taking its limit as *q* → 0. A non-negative least-squares regression was used to deconvolve the filter function in eqn (15) to recover the scattering spectrum, shown in Fig. 7.

**Fig. 7:**
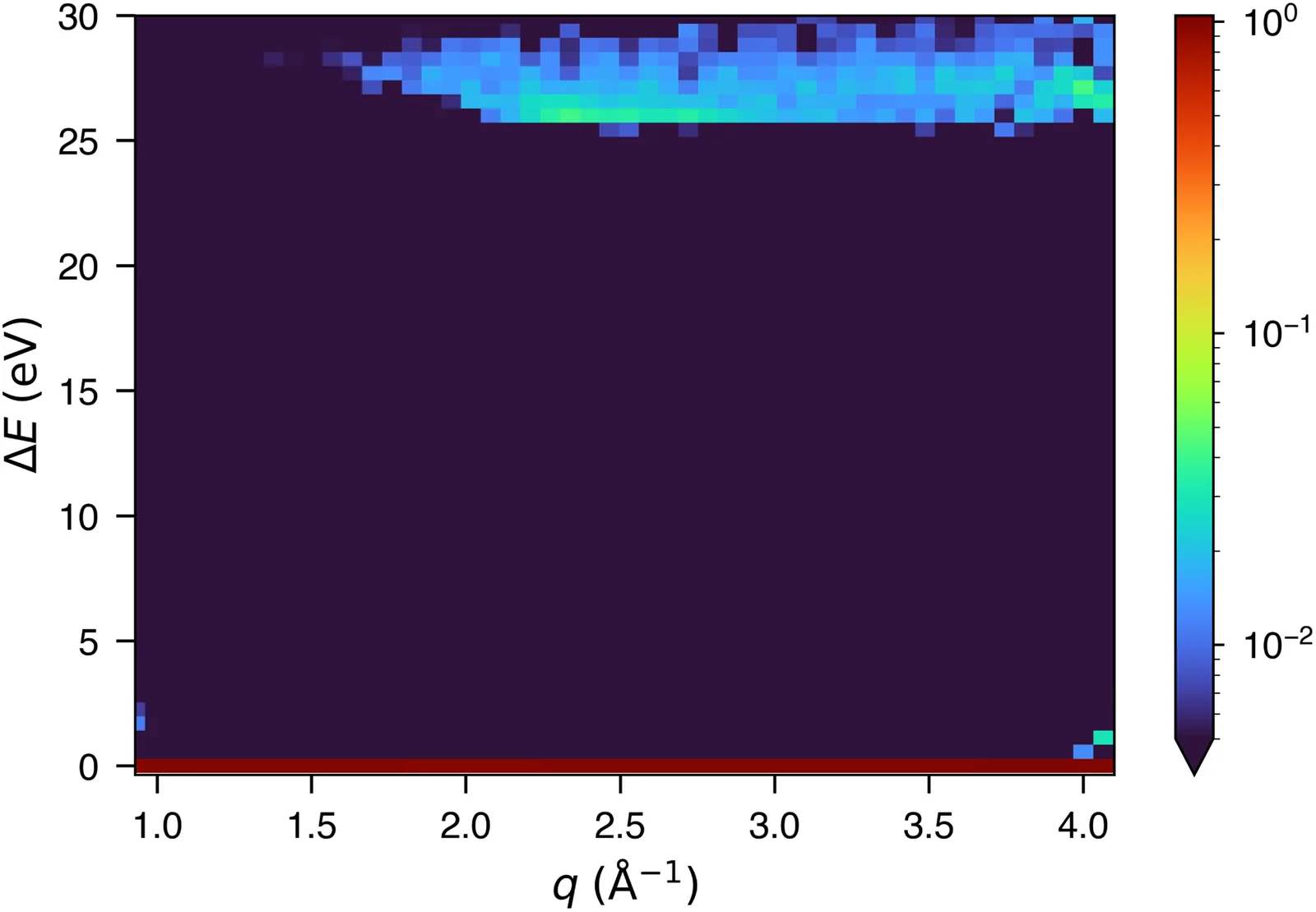
Recovered Bethe surface of SF_6_. Here, the dynamic structure factor has been normalized by the total scattering profile. The elastic scattering, seen at Δ*E* = 0 eV, encompasses a single bin, demonstrating the spectral resolution. The Compton scattering is summed into the feature near Δ*E* = 25 eV. The featureless region in between is reflective of insufficient statistics for recovering the onset of the Compton peak and the bound-to-bound inelastic transitions.

Due to the limited bandwidth of the X-ray pulses, the high-fidelity portions of the ***a*** vectors were limited. Following the deconvolution, this has the effect of combining any scattering with an energy loss greater than the bandwidth of the incident pulses into the final bins of the reconstructed scattering spectrum. Since the pulse bandwidth is ∼25 eV, the maximum energy loss that can be resolved is ∼25 eV. Therefore, any inelastic scattering that occurs at Δ*E* > 25 eV is compressed into the largest reconstructible energy loss bins.

The ghost-imaging technique successfully decomposes the total scattering into the DDSCS. The elastic scattering is almost completely contained within a single energy bin at Δ*E* = 0. In the inelastic regime, there is no visible signal except for a large feature above Δ*E* = 25 eV, representing a sum of the vast majority of inelastic scattering, resolved in *q*. This feature is attributed to Compton scattering. The Compton signal should start at ∼15 eV, close to the ionization potential of SF_6_. However, very few electrons contribute to the Compton scattering in the interval 15–25 eV, and thus the signal is much weaker compared to the sum of the Compton scattering signal above Δ*E* = 25 eV. In addition, transitions between bound-to-bound states should be present as *q*-dependent features in the range 1–15 eV as depicted in Fig. 1 for H_2_O. This signal should appear as a series of peaks that converge in intensity to the Compton scattering at 15 eV. However, due to the limitations of the experiment, we were unable to reconstruct the scattering spectrum with high enough signal-to-noise to recover these features. Evidently, these bound-to-bound inelastic features are dwarfed by the Compton scattering above Δ*E* = 25 eV.

While current generation XFELs are capable of delivering ∼10^12^ photons per pulse and thus allow for spectrally resolved X-ray scattering experiments, we are still limited to samples with large scattering cross sections, such as SF_6_. While this affords a high signal-to-noise ratio for the total scattering signal, it has the consequence of decreasing the fraction of individual inelastic elements with respect to the total X-ray scattering signal, consistent with Fig. 7.

Improvements can be made to the method. As stated in Section 2.3, fluorescence from the zinc foil needed to be accounted for. While the corrections did remove the majority of the impact, they may inflict new errors. Also, the Jungfrau-4M does have a rough energy sensitivity, but is not sensitive enough for separation of the zinc fluorescence from the scattering. In addition, individual photons incident on the detector can trigger more than one pixel, distributing their energy across them. Photon counting could be used to remedy this, but those algorithms are only effective when the number of photon strikes per pixel per image is ≲ 1. In this experiment, there are a large number of incident photons per pixel per image at low scattering angles and a small number at high scattering angles. This suggests a hybrid continuous/discrete approach, counting photon strikes to identify and filter out fluorescence counts at high scattering angles, while using the fluorescence method shown here in the continuous, low scattering angle regime.

## 4 Conclusions

We have demonstrated a spectral ghost-imaging technique that enables the model-independent spectral decomposition of the total X-ray scattering signal of gas-phase molecules using a conventional energy-integrating area detector at an XFEL. By exploiting the inherent shot-to-shot spectral stochasticity of SASE pulses in conjunction with a thin zinc foil acting as a spectral filter and a pre-specimen single-shot spectrometer, the scattering spectrum of SF_6_ was measured at the LCLS.

The methodology presented here is general and not limited to static systems. In a pump–probe experiment, the same spectral ghost-imaging approach could be applied to time-resolved X-ray scattering experiments, enabling simultaneous and accurate tracking of structural dynamics through the elastic scattering signal and the associated electronic spectrum *via* the inelastic scattering. Given the rich information about excited states that can already be inferred from elastic or total scattering alone,^47–50^ it is clear that such measurements would provide an unprecedented window into the coupled nuclear and electronic landscape that governs photochemical processes, charge transfer, and non-adiabatic transitions in general.

A key limitation of the present work is the counting statistics afforded by the 120 Hz repetition rate of the LCLS. The ghost-imaging reconstruction relies on correlating the stochastic spectral fluctuations across many pulses. The fidelity and precision of the reconstructions, particularly across the relatively weak bound-to-bound transition and onset of Compton scattering regions, is directly tied to the number of independent measurements. The upcoming LCLS-II-HE, operating at repetition rates of up to 1 MHz, will dramatically increase the rate of data collection and provide large improvements in counting statistics. This will not only allow for increased spectral resolution of the recovered DDSCS, but will make this technique more feasible for weakly scattering targets and time-resolved experiments where the available signal per time-delay bin is inherently less. These advances position spectral ghost imaging as a powerful and broadly applicable technique for the next generation of gas-phase X-ray scattering experiments at high-repetition-rate XFELs.

## Author contributions

Conceptualization: P. M. W. Formal analysis: D. J. R., B. H. S., A. M. C., P. Y. W., M. S., A. K., P. M. W. Funding acquisition: A. K., P. M. W. Investigation: D. J. R., A. N.-J., L. H., S. W. C., X. C., M. L., S. M., M. P. M., P. M. W. Methodology: P. M. W. Project administration: P. M. W. Resources: D. J. R., J. D. G., X. C., M. L., S. M., M. P. M., A. K., P. M. W. Software: D. J. R., A. M. C., P. Y. W., M. S., P. M. W. Supervision: D. J. R., A. K., P. M. W. Validation: D. J. R., B. H. S., A. M. C., M. S. Visualization: D. J. R., B. H. S., A. M. C., M. S. Writing – original draft: D. J. R., B. H. S., A. M. C., M. S., A. K., P. M. W. Writing – review & editing: D. J. R., B. H. S., A. M. C., M. S., A. K., P. M. W.

## Conflicts of interest

There are no conflicts to declare.

## Data Availability

Experimental data is available upon request. Raw data is stored at SLAC National Accelerator Laboratory, under the L10376 experiment.
